# Identification and Profiling of MicroRNAs During Embryogenesis in the Red Claw Crayfish *Cherax quadricarinatus*

**DOI:** 10.3389/fphys.2020.00878

**Published:** 2020-09-14

**Authors:** Yan Wang, Baojie Wang, Xuqing Shao, Mei Liu, Keyong Jiang, Mengqiang Wang, Lei Wang

**Affiliations:** ^1^CAS Key Laboratory of Experimental Marine Biology, Institute of Oceanology, Chinese Academy of Sciences, Qingdao, China; ^2^University of Chinese Academy of Sciences, Beijing, China; ^3^Shandong Cigna Detection Technology Co., Ltd., Qingdao, China; ^4^MOE Key Laboratory of Marine Genetics and Breeding, Ocean University of China, Qingdao, China; ^5^National Laboratory for Marine Science and Technology, Center for Marine Molecular Biotechnology, Qingdao, China; ^6^CAS Center for Ocean Mega-Science, Chinese Academy of Sciences, Qingdao, China; ^7^Laboratory for Marine Biology and Biotechnology, National Laboratory for Marine Science and Technology, Qingdao, China

**Keywords:** *Cherax quadricarinatus*, embryonic development, eye pigments forming stage, prepare-hatching stage, larvae, microRNA

## Abstract

MicroRNAs (miRNAs) are endogenous small non-coding RNAs that constitute a broad layer of gene regulation at both transcriptional and post-transcriptional levels from prokaryotes to eukaryotes. In embryonic development, they regulate the complex gene expression associated with the complexity of embryogenesis. There is little information about miRNAs in the red claw crayfish (*Cherax quadricarinatus*), an important commercial species and a potential biological model. In the present study, miRNAs and their target genes were identified during three embryonic developmental stages of *C. quadricarinatus*. Nineteen known miRNAs and 331 novel ones belonging to 50 miRNA families were obtained. A total of 113 differentially expressed miRNAs were identified, and 2,575 target genes were predicted, of which 1,257 were annotated. Additionally, 63 target genes of 9 miRNAs in *C. quadricarinatus* were found to be related to embryonic development. For example, miR-10 and its target genes may regulate the nervous system development and body segmentation and miR-2788 may regulate cell proliferation to impact embryonic development. Moreover, miR-28 (target gene *tutl*), miR-50 (target gene *fbx5*), and miR-1260b (target gene *sif*) may co-regulate eye development of embryonic *C. quadricarinatus*. These miRNAs together with their target genes constitute a network for regulating the development of tissues and organs in the embryo of *C. quadricarinatus*. Our results lay a foundation for further study on the fundamental molecular and developmental mechanism of crustacean embryogenesis.

## Introduction

Since Crick proposed the central dogma of molecular biology in 1958, research on genetic information has mainly focused on DNA and proteins. This is because the former acts as both storage and carrier of genetic information, while the latter is the expresser and executor of genetic information. However, in recent years it has been found that the major component of mammalian transcriptome is non-coding RNA (Okazaki et al., 2002). In other words, a considerable portion of the genetic information transmitted by DNA does not reach proteins and is only used by non-coding RNA, which means that a large amount of genetic information needs to be processed and transmitted after transcription and before translation. Therefore, the regulation of gene expression at the post-transcriptional level of microRNAs (miRNAs) requires further research, particularly since they are the most important post-transcriptional regulators discovered in recent years (Bartel, 2004).

MiRNAs are a class of small non-coding RNAs, composed of approximately 22 nucleotides (nt), that constitute a broad layer of gene regulation from prokaryotes to eukaryotes (Rupani et al., 2013). MiRNAs usually act as endogenous repressors of gene activity by repressing targeted gene translation and degrading target messenger RNA (mRNA) (He and Hannon, 2004). MiRNAs are essential for the normal development of plants and animals (Ebert and Sharp, 2012), and have a role in a wide variety of biological processes, including embryo formation, developmental patterning, cell proliferation, cell apoptosis, cell differentiation, and viral infection (Rhoades et al., 2002; Pernaute et al., 2011; Nguyen et al., 2017; Chen et al., 2018; Liang et al., 2018; Peng et al., 2018).

After the first miRNA was found in *Caenorhabditis elegans* in 1993 (Lee et al., 1993), large numbers of miRNAs have been subsequently discovered in a wide range of species using bioinformatics and experimental methods (Heimberg et al., 2010; Jima et al., 2010). With the development of next-generation sequencing (NGS) technology, large numbers of miRNAs have been obtained in crustaceans, such as *Portunus trituberculatus* (Chen et al., 2019), *Procambarus clarkia* (Yang et al., 2019), and *Litopenaeus vannamei* (Wang et al., 2019). These studies of miRNAs in crustaceans have been focused on the molecular basis of responses to environmental stimuli and the role in immune defense mechanisms (He Y. et al., 2015; Chen et al., 2019). Concerning development, miRNAs have been identified in *Macrobrachium olfersii* (Jaramillo et al., 2019), *Eriocheir sinensis* (He L. et al., 2015), and *P. trituberculatus* (Meng et al., 2018). However, information on the identification and function of miRNAs related to crustacean development is rare due to the diversity of this species.

The red claw crayfish (*Cherax quadricarinatus*) is a large and economically significant species in several countries, such as Australia, Mexico, Uruguay, and China (Macaranas et al., 1995), and is also increasingly used as a model organism in aspects of crustacean evolution and biology (Nguyen et al., 2016). Several studies have described the morphological and chronological characteristics of embryonic development in *C. quadricarinatus* (Jones, 1995; Levi et al., 1999; Meng et al., 2000; Yeh and Rouse, 2010). Its development process was divided into cleavage, blastula, gastrula, egg-nauplius stage, egg-metanauplius stage, eye pigments forming stage, and prepare-hatching stage based on the external morphological characteristics of an embryo (Meng et al., 2000). Nevertheless, few studies have been reported on the molecular mechanism of embryonic development of *C. quadricarinatus*. There was previously one report on the immune response against white spot syndrome virus (WSSV) infection in miRNA of *C. quadricarinatus* (Zhao et al., 2016), but the embryonic development of *C. quadricarinatus* miRNAs has not yet been described.

In the present study, we used the small RNA-Seq to identify miRNAs and acquire their expression profiles of *C. quadricarinatus* at three embryonic developmental stages, including eye pigment forming stage (EP), prepare-hatching stage (PH), and larvae (L). Differentially expressed miRNAs analysis was performed and their target genes were also predicted to examine the gene network involved in the regulation of embryonic development in *C. quadricarinatus*. This is the first systematic miRNA analysis of embryonic development stages in *C. quadricarinatus*. Our results revealed the characteristics and dynamics of miRNAs during embryonic development of *C. quadricarinatus* and lay a foundation for further study on the fundamental molecular and developmental mechanism of crustacean embryogenesis.

## Materials and Methods

### Sample Collection and Total RNA Extraction

The red claw crayfish were raised in a farm at Boxing, Shandong, China. Healthy ovigerous female crayfish were cultured in freshwater at 28°C. Embryos were collected at 20, 27, and 35 days after fertilization to represent different stages of embryo development, with three replications at each developmental stage. The sampled embryos were identified as in EP, PH, and L stages, respectively. A total of nine samples were flash-frozen in liquid nitrogen and then stored at −80°C until total RNA extraction. Total RNA was extracted from each sample using TRIzol reagent (Thermo Fisher Scientific, Waltham, MA, United States) according to the manufacturer’s instructions. The purity, concentration, and integrity of the RNA samples were tested using NanoDrop (Thermo Fisher Scientific, Waltham, MA, United States), Qubit 2.0 (Thermo Fisher Scientific, Waltham, MA, United States) and Agilent 2100 bioanalyzer (Agilent Technologies, Santa Clara, CA, United States). Only RNA samples with qualified OD 260/280 ≥ 1.8, OD 260/230 ≥ 1.0, total concentration ≥ 250 ng/μL, RIN number ≥ 8.0, and 28S/18S ≥ 1.5 were used for further sequencing.

### Small RNA Library Construction and Sequencing

For the samples up to standard, the amount of 1.5 μg was taken as the starting amount of total RNA sample, and the volume was supplemented to 6 μL with water. Nine small RNA libraries were constructed using the NEBNext^®^ Multiplex Small RNA Library Prep Kit for Illumina^®^ (New England Biolabs, Ipswich, MA, United States) according to the manufacturer’s recommendations. Initially, the small RNA was ligated with 3′ SR adaptor and 5′ SR adaptor using T4 RNA ligase. Then, the resulting samples were reverse transcribed into first-strand cDNA. Lastly, after PCR amplification, a gel purification was carried out to select sizeable fragments, which were purified to complete the construction of the library. Qubit 2.0 was used to detect the concentration of the library, which was diluted to 1 ng/μL. The insert size was detected by an Agilent 2100 bioanalyzer, and the effective concentration of the library was accurately quantified by quantitative real-time PCR (qPCR) to ensure the quality of the library. When the insert size was not greater than 320 bp and the effective concentration was greater than 1 ng/μL, the small RNA libraries were sequenced on an Illumina Hiseq 2500 platform and single-end reads with a sequencing read length of 50 nt were generated.

### Identification of miRNAs in *C. quadricarinatus*

The original image data file obtained by sequencing was transformed into the raw Reads by base calling. The Q-score (an integer mapping of the probability of base calling error) was Q30, that is, 1 misidentified base in 1000 bases. The raw reads generated from small RNA libraries were firstly processed through in-house perl scripts by excluding low quality reads, eliminating reads with 5′ primer contaminants, discarding reads without 3′ primer, and removing sequences smaller than 15 nt or longer than 35 nt. The final clean reads were obtained, and their length distribution was summarized.

The clean reads were aligned against the following databases: Silva^1^, GtRNAdb^2^, Rfam^3^, and Repbase^4^ by Bowtie (Langmead et al., 2009), to filter ribosomal RNA (rRNA), transfer RNA (tRNA), small nuclear RNA (snRNA), small nucleolar RNA (snoRNA), and other ncRNA. The remaining reads were regarded as unannotated reads and further alignment against reference sequences using Bowtie. Because of the lack of an annotated genome for *C. quadricarinatus*, the transcriptome of embryogenesis was used as the reference sequence. Reads aligned to the reference sequence were regarded as mapped reads and compared with the mature miRNAs against the miRBase database (v21) to detect known miRNA. The reads aligned to the mature miRNA sequences with no mismatches were regarded as known miRNAs. The novel miRNAs were predicted using miRDeep2 (Friedlander et al., 2008). The miRNAs were screened using RNAfold^5^ to predict the secondary structure and further verify the accuracy of the novel miRNAs.

### Differential Expression Analysis of miRNAs

Differential expression analysis of the three development stages was performed by using the DESeq (v1.18.0), which provides statistical methods for determining differential expression in digital gene expression data, using a model based on the negative binomial distribution. The miRNA expression quantity from the nine samples was counted and normalized by transcript per million (TPM = Read count/Mapped Reads × 1,000,000). The miRNAs with *p* < 0.05, False Discovery Rate (FDR) < 0.05, and fold-change ≥ 1.5 or fold-change ≤ 2/3 were assigned as differentially expressed. The fold-change represents the ratio of expression quantity between two groups. To display the expression profile, hierarchical clustering analysis of the differentially expressed miRNAs (DEMs) was performed in the form of a heat map.

### Target Genes Prediction

To understand the regulatory genes of DEMs in *C. quadricarinatus* embryos, miRNAs were analyzed using miRanda (Betel et al., 2008) and RNAhybrid (Friedlander et al., 2008), according to the transcriptome of embryogenesis to predict their target mRNAs. To gain further insight into the functions and classifications of the identified miRNAs target genes, targets were annotated based on the following databases: NCBI non-redundant protein sequences (NR)^6^; Swiss-Prot^7^; Gene Ontology (GO)^8^; Kyoto Encyclopedia of Genes and Genomes (KEGG)^9^; Clusters of Orthologous Groups (COG)^10^; EuKaryotic Orthologous Groups (KOG)^11^, Protein family (Pfam)^12^; and evolutionary genealogy of genes: Non-supervised Orthologous Groups (eggNOG)^13^.

### Verification of miRNAs by qPCR

To validate the expression levels of DEMs, eight DEMs were randomly selected to verify their relative expression by qPCR technique. Total RNA was extracted from the same samples as those used in Illumina sequencing. Tailing Poly (A) of miRNA, synthesis of first-strand cDNA and qPCR reactions was performed with the *TransScript*^®^ Green miRNA Two-Step qRT-PCR superMix (TransGen Biotech, Beijing, China). The miRNA forward primers were designed according to the miRNA Illumina sequencing data listed in Table 1. The reference gene U6 was used as an internal control. The qPCR reaction conditions were as follows: denaturation for 30 s at 94°C, followed by 40 cycles of 5 s at 94°C, and 31 s at 60°C. All reactions were performed with three biological replicates, and the relative miRNA expression was calculated using the Comparative C_T_ (2^–Δ^ ^Δ^ ^Ct^) method.

**TABLE 1:** Nucleotide sequences of primers used in this study.

Name	Sequence (5′–3′)
aca-miR-10a-5p-F	TACCCTGTAGATCCGAATTTGTG
aca-miR-29b-F	TAGCACCATTTGAAATCAGTG
bta-miR-1260b-F	ATCCCACCACTGCCACCA
hme-miR-2788-3p-F	CAATGCCCTTGGAAATCCCA
unconservative_c100602.graph_c1_20995-F	GCTGCTGCCTCCACT
unconservative_c100632.graph_c0_22409-F	AGACTGAGGGACTGACT
unconservative_c100988.graph_c1_35957-F	AAGTGAGGAGAGGCTGT
unconservative_c103160.graph_c3_133882-F	AATTGTTTTACATGATGGTAGG
U6-F	CGTGAAGCGTTCCATATTTTAA

### Statistical Analysis

The qPCR experimental data were reported as mean ± SD. Statistical analysis of qPCR results was performed by one-way analysis of variance (one-way ANOVA) using SPSS Statistic 19.0 software (SPSS, Chicago, IL, United States), and *p* < 0.05 were considered statistically significant.

## Results

### Output of Small RNA-Seq in *C. quadricarinatus*

**FIGURE 1:**
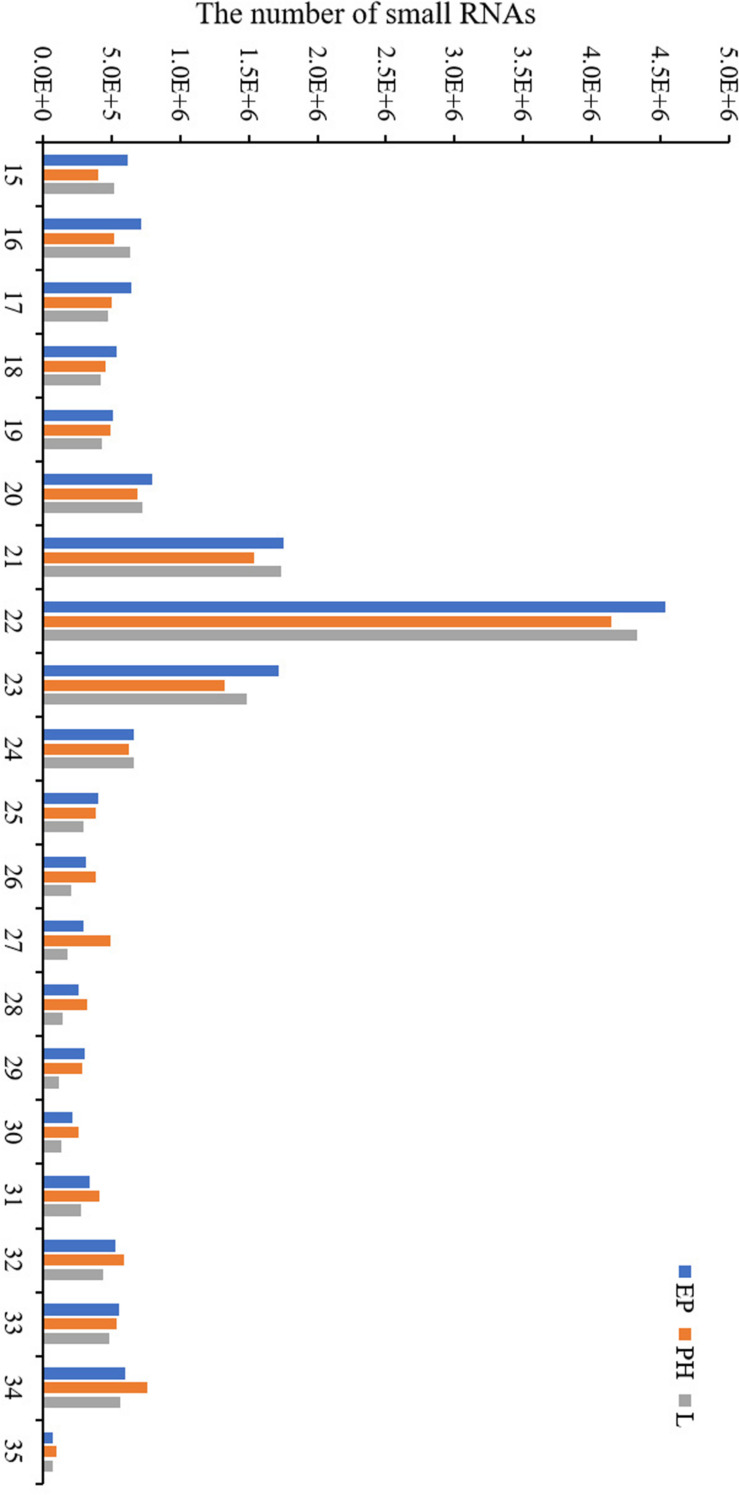
Length distribution of the small RNA obtained by high-throughput sequencing in the three libraries.

**FIGURE 2:**
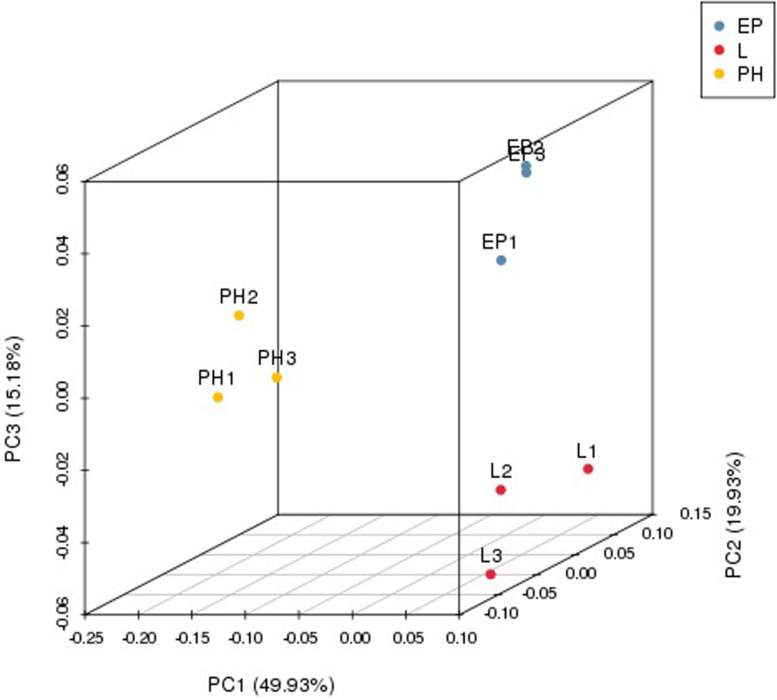
Principal component analysis (PCA) for the embryo samples.

Nine small RNA libraries were constructed from EP, PH, and L groups, and three different developmental stages of embryos. The original data set was deposited in the NCBI SRA database (Accession number PRJNA635700). A total of 13,728,979–23,185,872 clean reads, representing 690,686–1,683,669 unique sequences were obtained for each library (Table 2). These clean reads ranged from 15 to 35 nt, with the size distribution of clean reads is shown in Figure 1. The majority of these were 20–24 nt in length, with 22 nt the most abundant length, which is consistent with the typical size range for small RNAs generated by DICER. The clean reads were aligned against various databases and annotated into different RNA classes, meanwhile, 9,965,334–18,965,496 unannotated reads were obtained, which contained miRNA. We mapped a total of 666,815–1,710,191 reads by alignment with reference sequences (Table 2). The consistency of the sample collection and investigation of the miRNA relationship among embryos was evaluated by principal component analysis (PCA). PCA analysis revealed close clustering of biological replicates (Figure 2).

**TABLE 2:** The output of miRNA-seq.

Sample	Clean reads	Unannotated reads	Unique reads	Mapped reads	Mapped reads (+)	Mapped reads (−)
EP1	22,117,658	16,632,102	1,346,431	1,594,230	571,935	1,022,295
EP2	23,185,872	18,157,593	1,430,029	1,710,191	603,843	1,106,348
EP3	19,600,679	12,568,204	1,683,669	1,237,026	450,004	787,022
PH1	21,758,315	18,965,496	1,161,158	1,063,545	441,005	622,540
PH2	14,910,833	12,423,627	978,055	1,356,336	479,114	877,222
PH3	15,290,168	9,965,334	1,457,856	666,815	265,470	401,345
L1	13,728,979	12,037,383	690,686	1,378,103	392,866	985,237
L2	18,136,154	15,855,624	822,813	1,106,014	349,354	756,660
L3	20,004,305	16,844,070	1,026,800	1,123,473	410,410	713,063

### Identification of miRNAs in *C. quadricarinatus*

To identify known miRNAs in the nine libraries, the mapped reads were aligned with the miRNA homologs using miRBase. According to the biological characteristics of miRNA, miRDeep2 was used to predict novel miRNAs for the sequences without identifying known miRNAs. A total of 350 miRNAs were identified from all samples, including 19 known miRNAs and 331 predicted novel miRNAs. All the identified miRNAs belonged to 50 miRNA families (Supplementary Table S1). Among these 50 miRNA families, more than four-fifths consisted of only one member, such as miR-50, miR-467, miR-1293, and miR-6497. Nine miRNA families included multiple members, among them miR-10 was the most abundant family, comprising eight members, followed by miR-9193, miR-2162, miR-12, miR-29, miR-67, miR-980, miR-2788, and miR-8908.

### Differentially Expressed miRNAs in *C. quadricarinatus*

**FIGURE 3:**
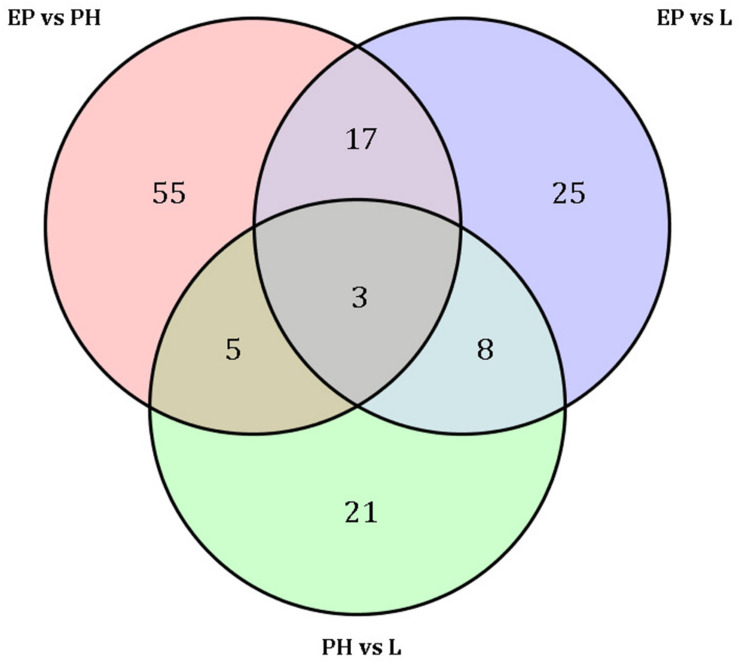
Venn diagram shows the number of differentially expressed miRNAs among three embryonic stages.

**FIGURE 4:**
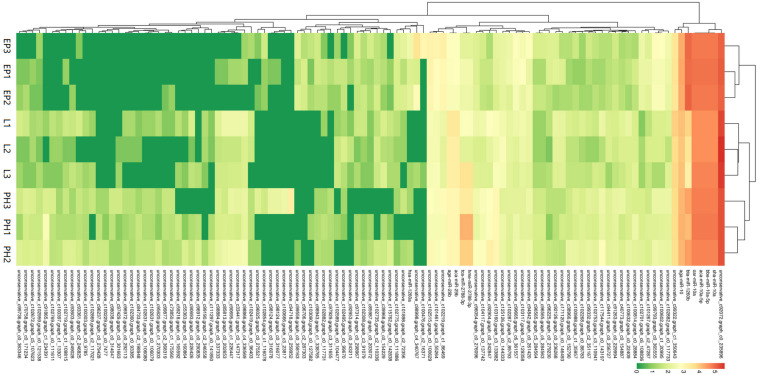
Cluster diagram of differentially expressed miRNAs. The abscissa represents different samples. The ordinate represents the different miRNAs. Log_10_ (TPM + 1) value was used for clustering. Red represents highly-expressed miRNAs and green represents low-expressed miRNAs.

A total of 80 miRNAs were identified as DEMs between EP and PH, of which 50 were significantly up-regulated (two were conserved and 48 were novel) and identified the miRNAs families miR-2788, miR-83, miR-2056, and miR-9193. The remaining 30 were significantly down-regulated (7 were conserved and 23 were novel) and identified four miRNAs families, miR-10, miR-1260a, miR-1260b, and miR-7594. Fifty-three miRNAs were identified as DEMs between EP and L, of which 30 were up-regulated (four were conserved and 26 were novel) and identified eight miRNAs families, miR-10, miR-28, miR-29, miR-50, miR-2788, miR-83, miR-2056, and miR-2284. The remaining 23 were down-regulated (2 were conserved and 21 were novel) and identified the miRNAs families miR-1260a, miR-1260b, and miR-9193. Comparing the L stage with the PH stage, 37 miRNAs were identified as DEMs, 11 up-regulated (2 were conserved and 9 were novel) and the identified miRNAs families were miR-28, miR-29 and miR-1011; as well as 26 down-regulated (3 were conserved and 23 were novel) and the identified miRNAs families were miR-1260b, miR-2788, and miR-2056. A total of 55, 25, and 21 DEMs were specific to EP vs. PH, EP vs. L, and PH vs. L, respectively. A total of 17 DEMs were overlapped between EP vs. PH and EP vs. L, and five DEMs were overlapped between EP vs. PH and PH vs. L, as well as eight DEMs were overlapped between EP vs. L and PH vs. L. Moreover, three DEMs were shared among the three comparisons (Figure 3). Hierarchical clustering analysis was performed on the DEMs and presented as heat maps (Figure 4). The heat maps showed that samples at three developmental stages were clustered respectively, indicating that miRNAs and expression patterns in different development stages were different.

### Prediction and Classification of miRNA Target Genes

To further understand the functions of DEMs at different stages in the embryo developmental of *C. quadricarinatus*, DEM target gene prediction was performed based on the transcriptome results of *C. quadricarinatus* embryogenesis. In total, 2,575 target genes were identified, of which 223 were target genes of known miRNAs and 2,402 were target genes of novel miRNAs. Among these target genes, 1,257 were annotated using BLAST against the following databases: NR (1,225 target genes annotated), Swiss-Prot (757 target genes annotated), GO (438 target genes annotated), KEGG (600 target genes annotated), COG (389 target genes annotated), KOG (859 target genes annotated), Pfam (1,031 target genes annotated), and eggNOG (1,105 target genes annotated), and 154 target genes were annotated by all these databases.

**FIGURE 5:**
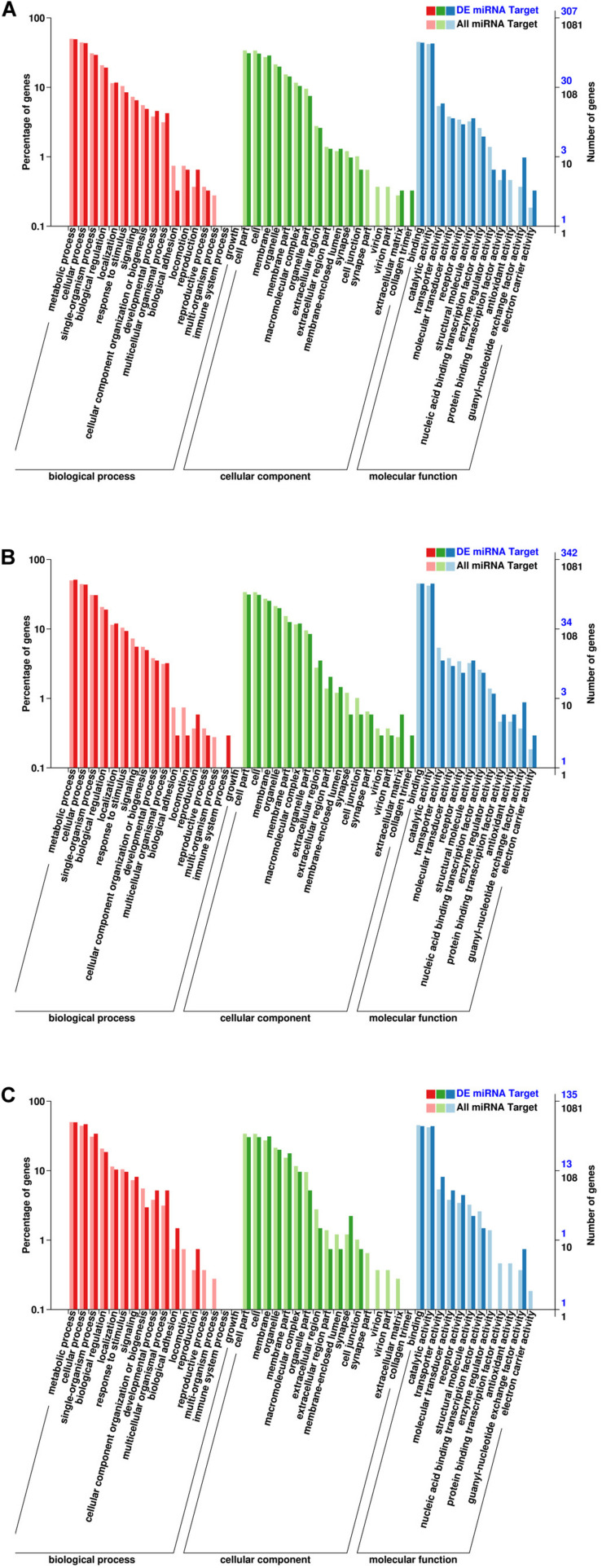
Gene ontology (GO) annotation of the DEMs. **(A)** EP vs. PH. **(B)** EP vs. L. **(C)** PH vs L. The abscissa represents the GO classification. The left ordinate represents the percentage of the number of genes, and the right ordinate represents the number of genes.

GO enrichment analysis was performed to identify the biological function of target genes in the three developmental stages (Figure 5). The results of the GO enrichment analysis of EP vs. PH showed that target genes were mostly enriched in the following categories: biological process (BP: metabolic process: 179 genes, cellular process: 155 genes, and single-organism processes: 126 genes), cellular component (CC: cell part: 142 genes, cell: 140 genes, and membrane: 123 genes), and molecular function (MF: binding: 165 genes, and catalytic activity: 159 genes). The results of the GO enrichment analysis of EP vs. L showed that target genes were mostly enriched in BP (metabolic process: 192 genes, cellular process: 173 genes, and single-organism processes: 130 genes), CC (cell part: 155 genes, cell: 154 genes, and membrane: 138 genes), and MF (binding: 169 genes, and catalytic activity: 158 genes). The results of the GO enrichment analysis of PH vs. L showed that target genes were mostly enriched in BP (metabolic process: 78 genes, cellular process: 54 genes, and single-organism processes: 28 genes), CC (membrane: 43 genes, cell part: 42 genes, and cell: 35 genes), and MF (binding: 60 genes, and catalytic activity: 52 genes).

**FIGURE 6:**
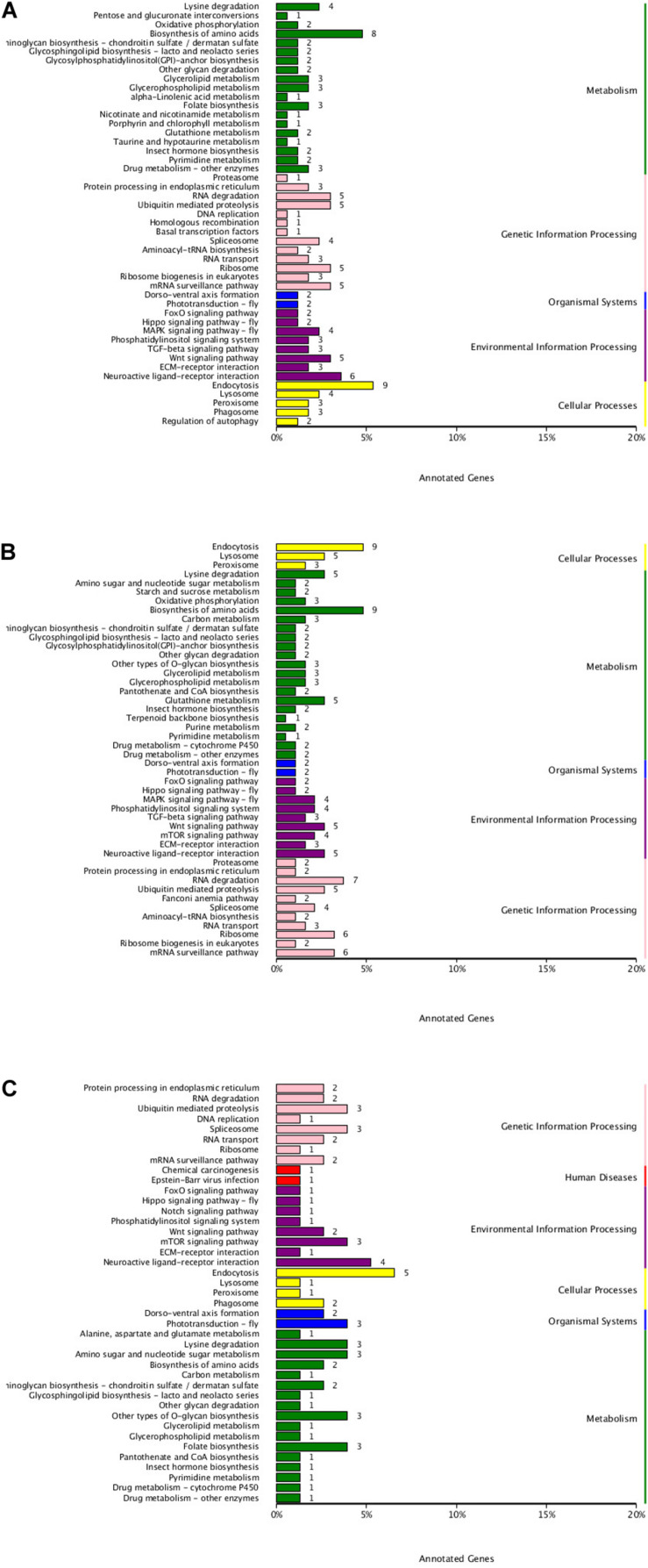
KEGG classification of DEMs. **(A)** EP vs. PH. **(B)** EP vs. L. **(C)** PH vs. L. The ordinate represents the name of the KEGG pathway. The abscissa represents the number of genes annotated into the pathway.

To understand the active pathways in developmental stages, target genes were further compared against the KEGG database (Figure 6). In total, 47 pathways were significantly enriched between EP and PH, including 19 metabolism pathways, comprising 45 genes, 13 genetic information processes pathways, comprising 39 genes, 8 environmental information processing pathways, comprising 28 genes, and five cellular processes, comprising 21 genes. In total, 46 pathways were significantly enriched between EP and L, including 21 metabolism pathways, comprising 58 genes, 11 genetic information processes pathways, comprising 41 genes, and nine environmental information processing pathways, comprising 32 genes. A total of 41 pathways were significantly enriched between PH and L, including 17 metabolism pathways, comprising 27 genes, eight environmental information processing pathways, comprising 14 genes, and eight genetic information processes pathways, comprising 16 genes.

Of these target genes with known functions, 63 were related to embryonic development. These 63 target genes may participate in neural, muscle, heart and eye development, embryo growth, as well as cell survival, proliferation, and migration during embryonic development of *C. quadricarinatus*, and were mediated by nine miRNAs (Tables 3, 4).

**TABLE 3:** Potential miRNA target genes related to embryonic development.

miRNA family	miRNA	Target gene or protein	Potential function of target genes
miR-10	aca-miR-10a-5p	Endoribonuclease dicer 1	Neural development as cell cycle dynamics in progenitor population, choice of cell fate, axon guidance, lamination, cell death or autophagy.
		AF4/FMR2 family member 4	Regulate embryonic cellularization, gastrulation, and segmentation
		ATP-dependent helicase brm	Suppresses the formation of ectopic neuroblasts
	c99322.graph_c1	Paramyosin, long form	Major structural component of muscle
		Neurogenic locus Notch	Regulate the development of the central and peripheral nervous system, eye, muscles, and segmental appendages
		Protein bicaudal D	Essential for differentiation
		Protein toll	Establishe dorsal-ventral polarity in the embryo
miR-28	c39968.graph_c0	Metal responsive transcription factor 1	Regulate embryonic growth
		Haemolymph juvenile hormone binding protein	Regulate embryogenesis
		Mediator of RNA polymerase II transcription subunit 15	Control early metazoan development
		PDZ domain	Particularly important in neurones
		Protein turtle	Eye development
		SWI/SNF-related matrix-associated actin-dependent regulator of chromatin subfamily B member 1 (SMARCB1)	Cell proliferation and differentiation
		Neural-cadherin	Participate in the transmission of developmental information
		Programmed cell death protein 2	Germinal center development
		Neurotrophin 1	Involved in the normal development of specific neurons at the neuromuscular junction
		Guanine nucleotide exchange factor MSS4 homolog	Cell polarity
		GTP-binding nuclear protein GSP1/Ran	Control of cell cycle
		Folliculin-interacting protein, middle domain	Energy and nutrient sensing
miR-29	c125126.graph_c0	Roundabout homolog 1	Neuronal development
		Vezatin	Morphogenesis of the embryo
		Structural maintenance of chromosomes protein	Ventral cord development
miR-50	c99190.graph_c2	Kinesin protein KIF11	Regulate embryonic growth
		Serine/threonine-protein kinase N	Regulate Rho-mediated dorsal closure during embryogenesis
		Suppressor of mec-8 and unc-52 protein	Affect multiple aspects of development
		Protein masquerade	Somatic muscle attachment and development of axonal pathways
		ubiA prenyltransferase domain-containing protein 1 homolog	Related to ectoderm development
		F-box/WD repeat-containing protein 5	Negatively regulates cell growth and proliferation in the eye
miR-1011	c102589.graph_c0	3-phosphoinositide-dependent protein kinase 1	Involved in axonal pathfinding and synaptogenesis
		Insulinoma-associated protein 1a	Play a role in neurogenesis and neuroendocrine cell differentiation
miR-1260a	hsa-miR-1260a	E3 ubiquitin-protein ligase HUWE1	Regulate neural differentiation
		Neurogenic locus notch homolog protein 1	Cell specification and differentiation
		Cytoskeleton-associated protein 5	Related to embryonic growth
		Glutamate receptor-interacting protein 1	Neural tube morphology
		Eukaryotic translation initiation factor 4H	Affect embryo growth
		Down syndrome cell adhesion molecule protein Dscam2	Play a crucial role in the development of visual system neurons
		Protocadherin Fat 4	Neuroprogenitor cell proliferation and differentiation
		Locomotion-related protein Hikaru genki	Play a role in the formation of functional neural circuits
		Cell adhesion molecule 4	Establishment of the myelin unit in the peripheral nervous system
		Longitudinals lacking protein	Axon growth and guidance in the central and peripheral nervous systems
		Wiskott-Aldrich syndrome protein family member 2	Affect embryo growth
miR-1260b	bta-miR-1260b	Protein prickle	Cell polarity
		E3 ubiquitin-protein ligase HUWE1	Regulates neural differentiation
		Nuclear receptor corepressor 1	Regulate embryo size
		Zinc finger homeobox protein 3	Embryonic central nervous system
		E3 ubiquitin-protein ligase hyd	Regulation of cell proliferation in germ cells
		Tyrosine-protein phosphatase non-receptor type 23	Related to embryonic growth
		F-actin-monooxygenase Mical	Play a key role in axon guidance and cell morphological changes
		Suppressor of cytokine signaling 5	Regulate epidermal growth factor receptor signaling
		Cytochrome P450 CYP302a1	Negatively regulates glial cell division in the embryonic midline
		Protein still life, isoforms C/SIF type 2	Required for eye development
		Protein charlatan	Required for correct development of the embryonic peripheral nervous system
		Nuclear hormone receptor FTZ-F1	Embryo development and differentiation
		Neurogenic locus protein delta	The correct separation of neural and epidermal cell lineages
		Inactive rhomboid protein 1	Cell survival, proliferation and migration
		Visual system homeobox 2	Role in the specification and morphogenesis of the sensory retina
		Homeobox protein homothorax	Required for patterning of the embryonic cuticle
		TGF-beta-activated kinase 1 and MAP3K7-binding protein 2	Heart development
		Paired amphipathic helix protein sin3a	Required for cortical neuron differentiation and callosal axon elongation
		Protein gawky	Required for completion of nuclear divisions during early embryonic development
		Longitudinals lacking protein	Axon growth and guidance in the central and peripheral nervous systems
miR-2788	hme-miR-2788-3p	Eukaryotic translation initiation factor 3 subunit C	Cell proliferation, including cell cycling, differentiation and apoptosis
miR-9193	c96225.graph_c0	Hemicentin	Promote cleavage furrow maturation during cytokinesis

**TABLE 4:** Specific information on potential miRNA related to embryonic development.

miRNA family	miRNA	EP vs. PH			EP vs. L			PH vs. L		
				
		*P*-value	FDR	log_2_FC	*P*-value	FDR	log_2_FC	*P*-value	FDR	log_2_FC
miR-10	aca-miR-10a-5p	0.00169	0.02318	−0.66978	0.02781	0.03953	−0.51171	0.87812	1.00000	0.25656
	c99322.graph_c1	0.26823	0.37967	0.24985	0.00172	0.03486	1.10479	0.01388	0.03906	0.94620
miR-28	c39968.graph_c0	0.87801	0.99452	−0.01783	0.00167	0.03569	1.96101	0.01662	0.02651	2.15004
miR-29	c125126.graph_c0	0.30339	0.67967	−0.27299	0.01805	0.02294	1.38835	0.00528	0.01734	1.76456
miR-50	c99190.graph_c2	0.02127	0.04651	2.95700	0.09299	0.14921	0.07736	0.00195	0.00244	2.11860
miR-1011	c102589.graph_c0	0.00024	0.00916	−2.54677	0.01173	0.03823	0.94279	0.02151	0.02493	3.53284
miR-1260a	hsa-miR-1260a	1.45E-05	0.00057	−3.99710	1.40E-06	1.22E-05	−4.86506	0.79769	1.00000	−0.64517
miR-1260b	bta-miR-1260b	5.93E-35	7.08E-33	−2.55536	9.81E-22	2.19E-19	−4.64798	0.00027	0.02402	−1.99602
miR-2788	hme-miR-2788-3p	0.03200	0.04142	4.99115	0.01197	0.02203	2.83243	0.02114	0.72569	−2.01119
miR-9193	c96225.graph_c0	0.00587	0.02196	−1.86154	0.00904	0.03345	−1.76111	1.00000	1.00000	0.11501

### Confirmation of miRNAs by qPCR

**FIGURE 7:**
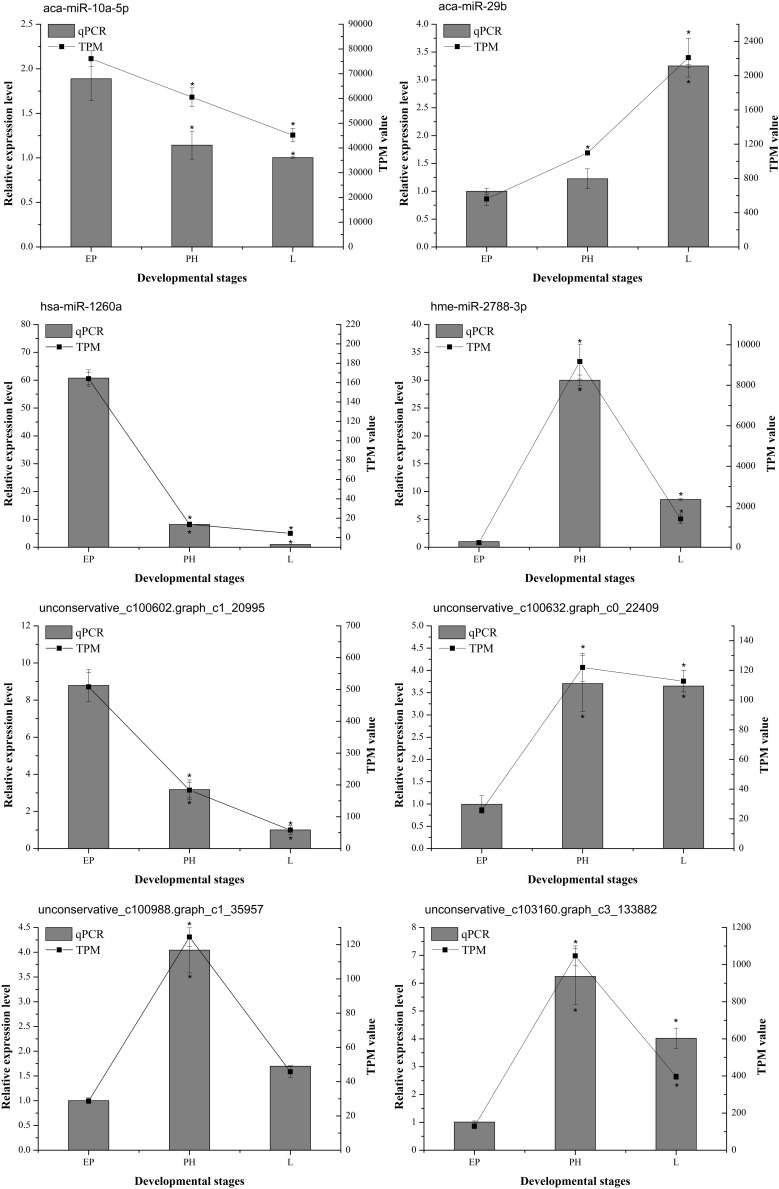
The expression profiles of selected miRNAs determined by miRNA-seq or qPCR. The abscissa represents the developmental stages. Columns and bars represent the means and standard deviation of qPCR relative expression levels, respectively (the ordinate at left). Lines represent the TPM value of miRNA-Seq (the ordinate at right). The asterisk represents statistically significant differences between EP and PH/L (*P* value was < 0.05).

To verify the effectiveness of the DEMs identified by miRNA-Seq, eight DEMs (aca-miR-10a-5p, aca-miR-29b, bta-miR-1260b, hme-miR-2788-3p, unconservative_ c100602.graph_ c1_ 20995, unconservative_ c100632.graph_ c0_ 22409, unconservative_ c100988.graph_ c1_ 35957, and unconservative_c103160.graph_c3_133882) were randomly selected to quantify their relative expression by qPCR. The results showed that these miRNAs exhibited similar expression patterns as seen with the high-throughput sequencing data (Figure 7), which further confirmed the accuracy and reliability of the miRNA expression changes detected using miRNA-Seq.

## Discussion

For the past few years, high-throughput sequencing has become an effective strategy for identifying miRNAs and studying their expression profiles in different developmental stages, tissues, and organs, as well as environmental conditions (Guo et al., 2016). MiRNAs regulate the development and physiological processes of different organisms, including embryonic, through degradation or translation inhibition of target genes (Moran et al., 2017). However, there are only a few reports on miRNA during embryonic development in crustaceans (Jaramillo et al., 2019). Furthermore, nothing about the role of miRNAs in embryonic development of *C. quadricarinatus* was known.

In the present study, three embryonic developmental stages of *C. quadricarinatus*, including the eye pigment forming stage, prepare-hatching stage, and larva, were selected by small RNA-Seq technology to identify miRNAs and acquire their expression profiles. In the previous reports, transcriptome libraries have been successfully used to search for novel miRNAs and predict target genes in some organisms (Niu et al., 2014; Wang et al., 2018). Although an annotated genome was lacking in *C. quadricarinatus* to identify miRNAs, the transcriptome of embryogenesis contributed to miRNAs recognition in this study. A total of 350 miRNAs (19 known miRNAs and 331 predicted novel miRNAs) belonging to 50 miRNA families were identified from all samples.

To understand the dynamic expression patterns of miRNA in different embryonic stages, we compared the miRNA expression variations and predicted their target genes. A total of 80 DEMs, 53 DEMs, and 37 DEMs were identified between EP and PH, EP and L as well as PH and L. Meanwhile, 2,575 target genes were predicted, of which 1,257 were annotated. Based on these data, 63 target genes for nine miRNAs in *C. quadricarinatus* were found to be related to embryonic development. The nine miRNAs are miR-10, miR-28, miR-29, miR-50, miR-1011, miR-1260a, miR-1260b, miR-2788, and miR-9193, and may participate in gene regulation related to neural, muscle, heart and eye development, embryo growth, as well as cell survival, proliferation, and migration during embryonic development of *C. quadricarinatus*.

Previous studies have shown that the miR-10 family directly regulates members of the Hox gene family, thus controlling the anterior-posterior axis pattern during *Nile tilapia* embryogenesis (Giusti et al., 2016). In our result, one of the target genes of miR-10 is AF4/FMR2 family member 4 (*lilli*). The gene *lilli* represents a novel pair-rule gene that acts in cytoskeleton regulation, segmentation, and morphogenesis during early *Drosophila* development (Tang et al., 2001). Another target of aca-miR-10a-5p (miR-10 family member) was the neurogenic locus notch gene (*notch*), which regulates the development of the central and peripheral nervous system, eye, muscles, and segmental appendages in *D. melanogaster* (Ramain et al., 2001; Portin, 2002). ATP-dependent helicase brm (*brm*) as the predicted target gene of c99322.graph-c1 (miR-10 family member) has been reported to suppress the formation of ectopic neuroblasts as part of the brm remodeling complex in *Drosophila* (Koe et al., 2014). Thus, we suggest a similar function for miR-10 in nervous system development and segmentation of *C. quadricarinatus* embryo. The expression of aca-mir-10a-5p was the highest in EP stage, while c99322.graph-c1 was the lowest in EP, indicating that EP might be a key period of neural development and segmentation.

In the embryonic development of *C. quadricarinatus*, miR-2788 target eukaryotic translation initiation factor 3 (*eif3*), which is involved in cell proliferation (Sigal et al., 2008). In *Heliconius melpomene*, miR-2788 target serine/threonine-protein kinase gene is involved in the development of wings and color patterning (Surridge et al., 2011). Similarly, in the present study, the serine/threonine-protein kinase N (*pkn*) has been described as a miR-50 target gene and is a Rho/Rac effector target required for dorsal closure during *Drosophila* embryogenesis (Lu and Settleman, 1999). Likewise, we propose miR-2788 functions in the regulation of cell proliferation and miR-50 functions in dorsal development in *C. quadricarinatus* embryo.

Twelve target genes of miR-28 related to embryonic development were predicted, and their functions were complex. MiR-28 has been identified in *Bos Taurus* blastocyst development (Goossens et al., 2013) and *Rattus norvegicus* (Zhu et al., 2016), and the function was not described. A target gene is turtle (*tutl*), which is involved in axonal targeting of the R7 photoreceptor in the developing eye (Cameron et al., 2013). The other two genes associated with eye development are the target gene of miR-50 and miR-1260b, miR-50 target gene *fbx5* (F-box/WD repeat-containing protein 5) negatively regulates cell growth, and proliferation in the wing and eye during *Drosophila* development (Moberg et al., 2001), on the contrary, miR-1260b target gene *sif* (protein still life, isoforms C/SIF type 2) is required for eye development of *Drosophila* (Ansar et al., 2018). These three miRNAs were expressed in all three stages of study, which may co-regulate the eye development of *C. quadricarinatus* embryonic.

There are many targeted genes of the same miRNA, and these targeted genes may have different functions. For example, besides the eye development mentioned above, miR-1260b also regulates nerve differentiation and heart development (Thienpont et al., 2010; Forget et al., 2014). There are also different miRNAs (miR-28, miR-50, and miR-1260b) that targeted different genes related to eye development. These miRNAs together with their target genes, form a network to regulate the development of *C. quadricarinatus* embryo.

## Conclusion

In this study, we undertook the first systematic miRNA analysis of embryonic development in *C. quadricarinatus*. We identified 19 known miRNAs and 331 predicted novel miRNAs during three developmental stages (eye pigment formation, prepare-hatching, and larval), further compared the differentially expressed miRNAs, and predicted their target genes. A total of 113 DEMs were identified, and 2,575 target genes were predicted, of which 1,257 were annotated. In addition, 63 target genes for nine miRNAs in *C. quadricarinatus* were found to be related to embryonic development. These miRNAs have different roles and together with their target genes constitute a network for regulating the development of tissues and organs in the embryo of *C. quadricarinatus*.

## Data Availability Statement

The original contributions presented in the study are publicly available. This data can be found here: https://www.ncbi.nlm.nih.gov/bioproject/PRJNA635700/ accession number PRJNA635700.

## Author Contributions

YW, MW, and LW conceived and designed the experiments. YW performed the experiments, analyzed the data, and drafted the manuscript. BW assisted with sample collection. XS, ML, and KJ assisted in part of the experiments. MW and LW participated in the coordination of the project and revised the manuscript. All authors read and approved the final manuscript.

## Conflict of Interest

XS was employed by the company Shandong Cigna Detection Technology. The remaining authors declare that the research was conducted in the absence of any commercial or financial relationships that could be construed as a potential conflict of interest.

## Footnotes

1 https://www.arb-silva.de/

2 http://gtrnadb.ucsc.edu/

3 http://rfam.xfam.org/

4 https://www.girinst.org/

5 http://rna.tbi.univie.ac.at/cgi-bin/RNAWebSuite/RNAfold.cgi

6 ftp://ftp.ncbi.nih.gov/blast/db/

7 http://www.uniprot.org/

8 http://geneontology.org/

9 http://www.genome.jp/kegg/

10 http://www.ncbi.nlm.nih.gov/COG/

11 http://www.ncbi.nlm.nih.gov/KOG/

12 http://pfam.xfam.org/

13 http://eggnog.embl.de
