# Supplementary material for: Identification and Profiling of MicroRNAs During Embryogenesis in the Red Claw Crayfish Cherax quadricarinatus
Source: Front Physiol. 2020 Sep 14;11:878. doi: 10.3389/fphys.2020.00878 (PMC7521159; doi:10.3389/fphys.2020.00878)
Supplement: Supplementary file 1 [file Table_1.docx]

Table S1 Identification of miRNA families in *C. quadricarinatus*

|  | **miRNA** | **miRNA family** |
| --- | --- | --- |
| known miRNAs | rno-miR-466b-3p | mir-467 |
|  | aga-miR-10 | mir-10 |
|  | bta-miR-1260b | mir-1260b |
|  | aga-miR-307 | mir-67 |
|  | hme-miR-2788-3p | mir-2788 |
|  | tca-miR-2788-3p | mir-2788 |
|  | sha-miR-10a | mir-10 |
|  | tur-miR-307-3p | mir-67 |
|  | aca-miR-29b | mir-29 |
|  | bbe-miR-10a-5p | mir-10 |
|  | aae-miR-12-5p | mir-12 |
|  | tur-miR-12a-5p | mir-12 |
|  | bmo-miR-10-3p | mir-10 |
|  | lmi-miR-10-3p | mir-10 |
|  | oar-miR-10a | mir-10 |
|  | age-miR-29b | mir-29 |
|  | hsa-miR-1260a | mir-1260a |
|  | aca-miR-10a-5p | mir-10 |
|  | tca-miR-12-5p | mir-12 |
| novel miRNAs | unconservative_c99190.graph_c2_346558 | mir-50 |
|  | unconservative_c88783.graph_c1_214817 | mir-8908 |
|  | unconservative_c39968.graph_c0_154229 | mir-28 |
|  | unconservative_c101462.graph_c2_55293 | mir-6497 |
|  | unconservative_c91294.graph_c0_230161 | mir-2513 |
|  | unconservative_c98499.graph_c2_326048 | mir-2162 |
|  | unconservative_c91099.graph_c0_228923 | mir-1293 |
|  | unconservative_c91122.graph_c0_229042 | mir-148 |
|  | unconservative_c99362.graph_c1_352050 | mir-6790 |
|  | unconservative_c99322.graph_c1_350643 | mir-10 |
|  | unconservative_c99400.graph_c1_353286 | mir-2162 |
|  | unconservative_c59423.graph_c0_165794 | mir-279 |
|  | unconservative_c99840.graph_c2_367043 | mir-785 |
|  | unconservative_c92904.graph_c0_242847 | mir-2056 |
|  | unconservative_c96225.graph_c0_279230 | mir-9193 |
|  | unconservative_c100510.graph_c3_17639 | mir-9193 |
|  | unconservative_c74864.graph_c0_176791 | mir-252 |
|  | unconservative_c102308.graph_c3_91079 | mir-7594 |
|  | unconservative_c84289.graph_c1_196922 | mir-980 |
|  | unconservative_c87561.graph_c0_208604 | mir-2058 |
|  | unconservative_c38908.graph_c0_152363 | mir-9193 |
|  | unconservative_c98945.graph_c3_338818 | mir-493 |
|  | unconservative_c102257.graph_c1_88237 | mir-2162 |
|  | unconservative_c102769.graph_c0_111611 | mir-83 |
|  | unconservative_c84289.graph_c1_196921 | mir-980 |
|  | unconservative_c115762.graph_c0_142838 | mir-2284 |
|  | unconservative_c90178.graph_c0_223452 | mir-3 |
|  | unconservative_c95451.graph_c0_268472 | mir-2162 |
|  | unconservative_c88783.graph_c1_214818 | mir-8908 |
|  | unconservative_c72127.graph_c0_173296 | let-7 |
|  | unconservative_c102589.graph_c0_104477 | mir-1011 |
|  | unconservative_c98786.graph_c0_334440 | mir-754 |
|  | unconservative_c84515.graph_c0_197906 | mir-154 |
|  | unconservative_c38908.graph_c0_152364 | mir-9193 |
|  | unconservative_c93332.graph_c0_246552 | mir-95 |
|  | unconservative_c104926.graph_c0_138808 | mir-7398 |
|  | unconservative_c99808.graph_c0_365947 | mir-9189 |
|  | unconservative_c38491.graph_c0_150900 | mir-210 |
|  | unconservative_c79373.graph_c0_184484 | mir-193 |
|  | unconservative_c101817.graph_c0_69073 | mir-77 |
|  | unconservative_c86404.graph_c0_204106 | mir-237 |
|  | unconservative_c98404.graph_c0_323252 | mir-544 |
|  | unconservative_c86404.graph_c0_204108 | mir-4178 |
|  | unconservative_c102711.graph_c2_109329 | mir-17 |
|  | unconservative_c125126.graph_c0_144333 | mir-29 |
|  | unconservative_c116936.graph_c0_143082 | mir-584 |
|  | unconservative_c101772.graph_c4_67278 | mir-1538 |
|  | unconservative_c102205.graph_c0_85681 | mir-9193 |
|  | unconservative_c98766.graph_c0_333775 | mir-9193 |
|  | unconservative_c101661.graph_c3_62940 | mir-8499 |
|  | unconservative_c100899.graph_c4_32309 | mir-450 |
|  | unconservative_c92408.graph_c0_238501 | mir-34 |
|  | unconservative_c38000.graph_c0_149649 | mir-724 |
|  | unconservative_c99240.graph_c1_348079 | mir-219 |
|  | unconservative_c56189.graph_c0_161780 | mir-2162 |
